# The molecular mechanisms of crista formation: how mitochondria give themselves breathing room

**DOI:** 10.1042/BST20260167

**Published:** 2026-08-27

**Authors:** Lilia Colina-Tenorio, Martina Bohuslavová, Alexander W. Bruce, Hassan Hashimi

**Affiliations:** 1 Department of Molecular Biology and Genetics, Faculty of Science, University of South Bohemia, České Budějovice, Czechia; 2 Institute of Parasitology, Biology Centre, Czech Academy of Sciences, České Budějovice, Czechia

**Keywords:** ATP synthase, cristae, dynamin-related protein, MICOS, mitochondria, oxidative phosphorylation

## Abstract

Cristae are mitochondrial subcompartments that give the organelle its distinctive appearance. More significantly, mitochondria are the proverbial powerhouses as cristae house the molecular machinery underlying cellular respiration, a process that converts carbon sources into ATP by chemiosmosis. The form of cristae is invariably connected to their bioenergetic function. Here, we review our current understanding of the molecules underpinning crista formation. Not surprisingly, respiratory chain multiprotein complexes are involved in crista formation, with F_1_F_O_-ATP synthase dimers being eminent membrane sculptors. But crista formation also requires factors that are not directly part of the respiratory chain. The most ancient is the MICOS complex, which delineates the subcompartment and acts as a hub for crista biogenesis. The mitochondrial inner membrane (IM), from which cristae emerge, is remodelled by different dynamin-related proteins in animals and fungi. Cardiolipin is an integral component of the membranous fabric of the IM. To begin to grasp general design principles underlying crista formation, we synthesize findings from canonical animal and yeast experimental models with those from diverse protists and other eukaryotes. However, how these molecules are orchestrated during crista formation remains a hidden piece in our understanding of how cells differentiate in specialized forms. We highlight the few knowns about crista formation in a handful of organisms to guide research into the many unknowns about how complex subcompartments represented by mitochondrial cristae are formed.

## Introduction

**Figure 1:**
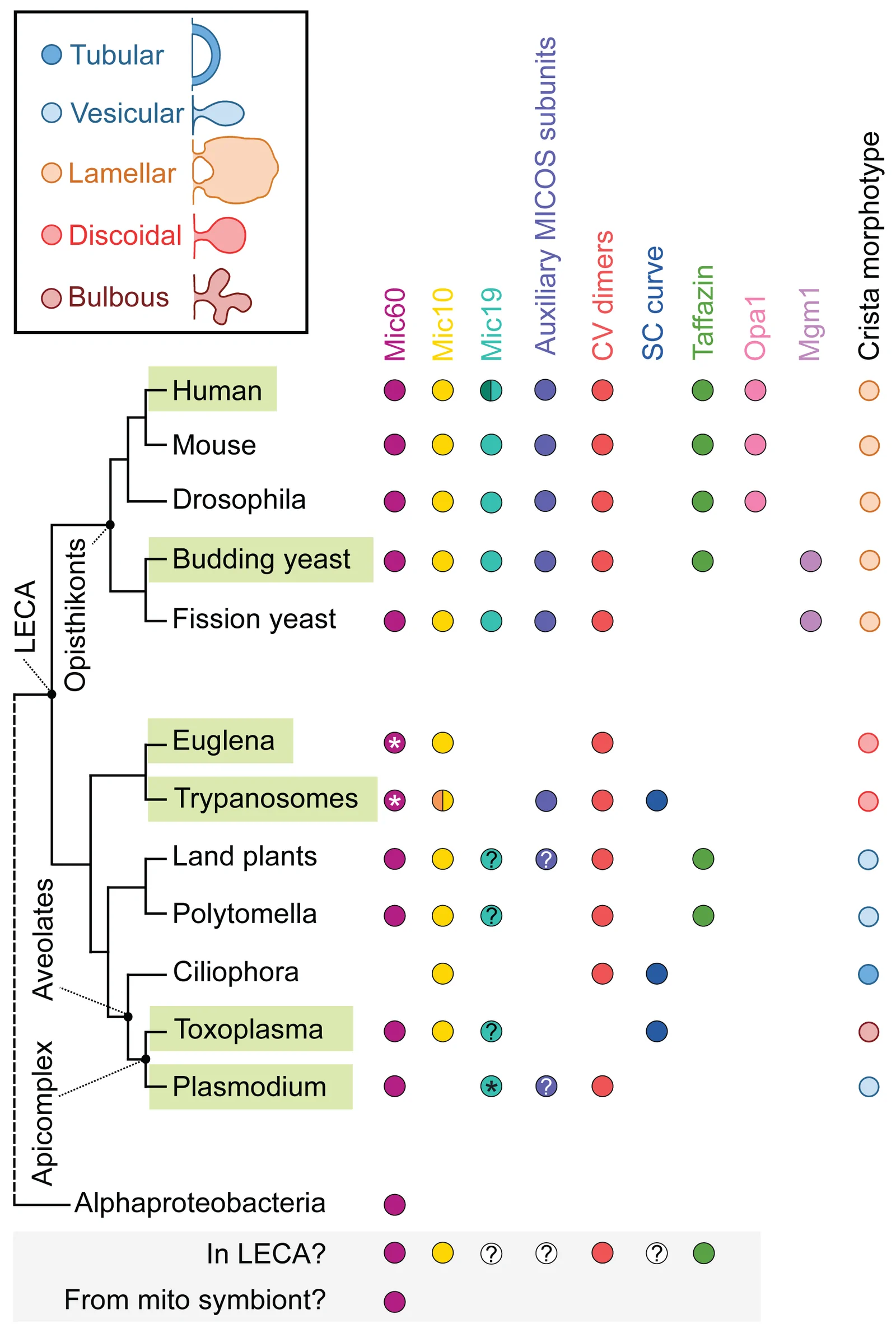
The phylogenetic distribution of crista morphotypes and selected crista formation factors in the organisms discussed in this review The eukaryotic lineages discussed in this review are shown on an illustrative tree in relation to alphaproteobacteria [118]. The position of the LECA at the root of the eukaryotic tree plus the roots of specific taxonomic groupings are marked by back circles. Organisms highlighted with green are depicted in Figure 2. Dot columns indicate the verified presence of crista-shaping proteins/complexes listed in the top row. CV, F_1_F_O_-ATP synthase; SC curve, experimentally verified membrane curvature induced by OXPHOS supercomplexes; *, highly diverged forms of Mic19 and Mic60, the latter of which are named Mic34 and Mic40; ?, too few described cases to conclude if present or absent from LECA. The split circle indicates presence of paralogs. Auxiliary MICOS subunits identified directly or suggested by size of MICOS complex (?). The two shaded rows at the bottom indicate presence in LECA inferred from wide phylogenetic distribution (In LECA?) and if the gene encoding the protein was inherited from the proto-mitochondrial symbiont (From mito symbiont?), as inferred from presence in extant alphaproteobacteria. Top left inset, crista morphotypes depicted in tree and discussed in review. Morphotypes assigned as previously [4,83].

Cristae are a trademark character of mitochondria, these invaginations of the inner membrane (IM) were observed before the organelle was known to harbour its own genome [1]. Mitochondrial DNA and the capacity to form cristae are also linked as traits that were directly inherited from the alphaproteobacterial symbiont that engendered mitochondria [2,3]. After ∼2 billion years of evolution, cristae have become highly diverse and dynamic structures present in all eukaryotes capable of aerobic respiration [4]. While cristae display different morphologies across eukaryotic lineages (Figure 1), certain key aspects are common: they are connected to the inner boundary membrane (IBM) by narrow membrane regions called crista junctions (CJs) [5], they house the complexes that mediate oxidative phosphorylation (OXPHOS) [6,7], and they constitute bioenergetic sub-compartments within mitochondria [8].

The molecular mechanism(s) that guide the biogenesis of mitochondrial cristae are still mostly unknown. Even though several proteins involved in cristae morphogenesis have been identified, how their different activities are coordinated during crista formation is far from understood. Here, we summarize our current knowledge about crista-shaping molecules, gained predominantly from the fungal and animal experimental models that belong to the Opisthokonta lineage of eukaryotes (Figure 1). However, orthologs of some of these molecules are found in other eukaryotic groups comprising protists and multicellular organisms such as land plants, sometimes diverged beyond initial recognition. Thus, contrasting information from all these organisms will serve the ultimate aim of proposing general design principles underlying crista formation in all eukaryotes.

## Molecules underlying cristae morphogenesis in opisthokonts

**Figure 2:**
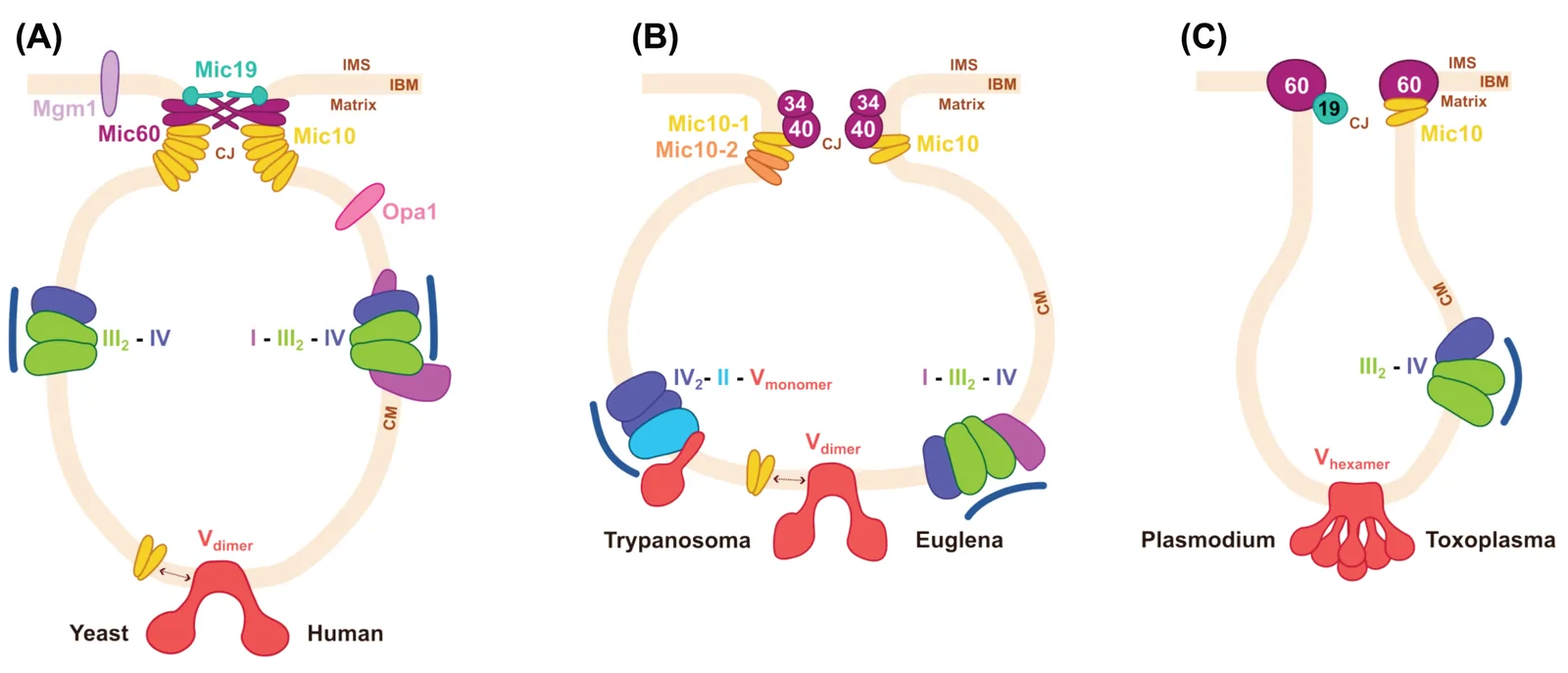
Crista-shaping proteins across eukaryotic lineages The mitochondrial IM organized as: (**A**) Lamellar cristae in yeast (left side) and humans (right side). MICOS core subunits (Mic60 tetramer and Mic10 oligomers) plus Mic19 are depicted. Respiratory supercomplexes in the crista membrane (CM) are shown as found in yeast and humans; the blue line indicates a flat region of the CM. Dimeric ATP synthase (labeled V dimer) is shown at the crista tip and cross-talk with Mic10 in yeast is represented with a double-headed arrow. (**B**) Discoidal cristae in Trypanosoma (left side) and Euglena (right side). The Mic10 paralogs (Mic10-1 and Mic10-2) found in Trypanosoma, as well as the mitofilin domain-containing proteins Mic34 and Mic40, are depicted. Distinct associations of respiratory complexes found in each organism are shown and their contribution to membrane curvature is indicated with a blue line. Cross-talk between ATP synthase (labeled V dimer) and Mic10-1 in Trypanosoma is represented as in panel (A). (**C**) Tubular cristae in Plasmodium (left side) and Toxoplasma (right side). Mic60 and the diverged version of Mic19 are shown for Plasmodium. The respiratory supercomplex of *T. gondii* and its contribution to membrane curvature is indicated as in panel (B). The ATP synthase hexamer (labeled V hexamer) is depicted at the crista tip. In panels (A), (B), and (C): IBM, inner boundary membrane; IMS, intermembrane space.

MICOS (mitochondrial contact site and cristae organizing system) is an oligomeric complex that was first found localized at CJs of yeast [9–12], and later found to fulfil a similar role in mammals [13,14]. The complex is divided into two membrane-anchored subcomplexes named after the core subunits Mic60 and Mic10, which are the most widely conserved and thought to be present in the last eukaryotic common ancestor (LECA) [15,16] (Figures 1 and 2A). Opisthokonts acquired additional MICOS subunits for a total of six proteins in *Saccharomyces cerevisiae* and seven in animals (Figure 1). Mic60 is not just the oldest MICOS subunit, but may be one of the first crista-shaping proteins, as it was likely inherited from the proto-mitochondrial endosymbiont [2,3], thus predating most of the molecules to be discussed here.

Yeast Mic60 and Mic10 have membrane shaping capacity *in vitro* [17–19], and deletion of either results in aberrant cristae that are detached from the IBM [13,20,21]. All the Mic60 proteins studied so far contain a central elongated coiled-coil region and a conserved mitofilin domain at their C-termini [15,16,22]. The latter facilitates Mic60’s membrane binding, an activity that is regulated by a soluble coiled-coil-helix-coiled-coil-helix (CHCH) protein named Mic19 [18]. Structural studies of the subcomplex comprising Mic60 and Mic19 suggest a role in stabilizing and controlling CJ constriction [23,24]. In addition, Mic60 associates with import machineries Translocase of the Outer Mitochondrial Membrane (TOM) and Sorting and Assembly Machinery (SAM) in the outer membrane, as well as with Translocase of the Inner Mitochondrial Membrane 22 (TIM22) in the IM, thus establishing outer/IM contact sites and assisting in the import of specific mitochondrial proteins [11,25–28]. The supercomplex comprised of MICOS and SAM is particularly stable and is called the mitochondrial intermembrane space (IMS) bridging complex [16,27]. Beyond its role in IM organization, MICOS also promotes respiratory complex assembly in yeast [12,29,30], an important contribution to crista biogenesis by incorporation of the machinery needed for OXPHOS.

Mic10 is a small protein made up of two transmembrane domains (TMDs) separated by a short loop, likely forming a wedge-like shape [17,20,31]. Mic10 homo-oligomerization has been proposed to confer membrane curvature at the CJs [17,20], thus acting synergistically with the Mic60 subcomplex. Mic10 oligomerization is controlled by apolipoprotein-like (ApoL) subunits of the Mic10 subcomplex [32], potentially adding another layer of regulation in the construction of CJs by MICOS. In fission yeast, the MICOS complex has been stripped down to Mic10, an ApoL subunit, Mic19 and Mic60, suggesting these subunits encapsulate the core activities of opisthokont MICOS [33].

In addition to fulfilling its role in producing ATP during OXPHOS, F_1_F_O_-ATP synthase has emerged as another major crista-shaping factor [34]. Remarkably, these two functions are uncoupled from each other. Promotion of local curvature at cristae tips depends solely on the dimerization of F_1_F_O_-ATP synthase (Figure 2A). When these dimers are disrupted, the enzymatic activity persists, but cristae are drastically altered into multi-layered rings resembling an onion in transmission electron micrographs [35]. In opisthokonts, F_1_F_O_-ATP synthase dimers assume a V-like shape emanating from the interaction interface between two membrane-embedded F_O_ sections [36].

Individual subunits e and g from each F_O_ monomer form homodimers that represent the interaction interface. While each dimer pinches a small part of the crista membrane (CM), the dimers assemble into rows that are responsible for positive curvature at crista rims [36,37]. Dimerization is exclusive to mitochondrial F_1_F_O_-ATP synthases and is a phenomenon observed in all lineages investigated thus far [34], indicating that this mechanism of crista shaping was already present in LECA [38,39], but evolved subsequent to acquisition of Mic60. Nevertheless, cross-talk between F_1_F_O_-ATP synthase and MICOS via a pool of free Mic10 has emerged to coordinate their respective roles in crista formation and function [40].

Given the recurrence of membrane bending in crista formation, it is not surprising that dynamin superfamily proteins, which oligomerize and hydrolyse GTP to remodel membranes [41], would be among key crista-shaping factors. Indeed, two such proteins stand out as key players in IM dynamics: dynamin-related protein Opa1 (optical atrophy 1) in animals [42,43] and Mgm1 (mitochondrial genome maintenance 1) in fungi [44,45]. However, unlike MICOS and F_1_F_O_-ATP synthase dimers, which have radiated from LECA throughout eukaryotes, Opa1 and Mgm1 are found almost exclusively in opisthokonts [46,47]. These proteins are anchored to the IM but in their processed, short form they are found in the IMS (GTPase domains extended toward the IMS). Both Opa1 and Mgm1 mediate IM fusion to drive this aspect of mitochondrial dynamics in their respective organisms [48–50] (Figure 2A). However, only Opa1 directly modifies cristae *in situ* [51], acting as a key regulator of cytochrome c release from the crista lumen to induce apoptosis [49]. Opa1 engages in cross-talk with Mic60 and several other MICOS subunits to coordinate their respective CM shaping activities [52,53]. In contrast, Mgm1 indirectly influences crista morphology via its IM fusion activity [54], and therefore does not interact with MICOS [14,55]. In fungi, except curiously the budding yeast *S. cerevisiae*, a dynamin-related protein named Mmc1 interacts with Mgm1 to generate and maintain cristae morphology, and it oligomerizes on the matrix side of the CM via an interaction with the Mic10 subcomplex [33]. This mechanism is believed to statically stabilize cristae, as Mmc1 cannot undergo conformational changes needed for membrane remodelling due to a loss of GTPase activity.

The localization and organization of phospholipids within the mitochondrial membranes influences both the shape and the function of mitochondria [56,57]. Here, we will concentrate on the non-bilayer phospholipids that form curved membranes [58]. Cardiolipin (CL) is mitochondria-specific and concentrated in the IM, where it fulfils many roles. Importantly, CL intercalates into OXPHOS super-complexes in both yeast [59,60] and mammals [61,62], and it is also important for the stability of the MICOS complex [21], specifically that of the Mic10 oligomers in yeast [32]. Phosphatidylethanolamine (PE) is a non-bilayer forming lipid that contributes to membrane curvature in mitochondria [63]; inhibition of its biosynthesis dramatically alters cristae morphology, mitochondrial function, and is embryonic lethal [64,65]. In yeast, depletion of both CL and mitochondrial PE is synthetically lethal [66]. These observations suggest there might be a specific need for negative lipid curvature in the mitochondrial IM [67].

Regarding the shaping of cristae, the direct contribution of CL remains puzzling. Deficiency in the acyltransferase Tafazzin, which catalyzes changes to the fatty acyl side chains of CL, leads to crista morphology defects [68]. This may indicate that CL alone promotes IM curvature, an effect that compensates for a decrease in spontaneous curvature due to oversaturation of fatty acyl side chains in all phospholipid classes [67]. Furthermore, Tafazzin deficiency also affects assembly of OXPHOS super-complexes [68,69], complicating interpretation of CM defects. So far, molecular dynamic simulations support the hypothesis that CL headgroup charge and the overall composition of the phospholipid bilayer are more critical to intrinsic curvature than the composition of their fatty acyl side chain [63,70].

## Molecules underlying cristae morphogenesis outside of opisthokonts

As evidenced by the wealth of knowledge described above, much is known about crista-shaping molecules in opisthokont experimental models. However, these data should be considered under the lens of evolution to truly understand general design principles that underlie crista formation. Moreover, the varied crista morphology observed across eukaryotes (Figure 1), which probably reflects the presence of divergent IM machineries and unique adaptations, remains unexplained [4]. In this chapter, we compile current knowledge about the factors underlying crista morphogenesis in non-opisthikonts.

MICOS appears to be a large complex in land plants [71] and the parasitic protists *Plasmodium falciparum* [72] and *Trypanosoma brucei* [31,73,74]. Unlike other apicomplexan parasites like *Toxoplasma gondii*, *P. falciparum* has lost Mic10 [15,16,72]; nevertheless, CJ formation in Plasmodium still requires Mic60 and a highly diverged Mic19 ortholog [72] (Figure 2C). This suggests that CJ formation is a fundamental function of MICOS dating back to LECA.

Trypanosomes bear the most studied MICOS complex outside of opisthokonts, and perhaps the strangest iteration [74]. In stark contrast with opisthokont MICOS, the trypanosomal complex is subdivided into a membrane-integral Mic10 complex and a subcomplex comprised exclusively of soluble proteins that are peripheral to the IM [73]. This peripheral complex is built around two cryptic mitofilin superfamily proteins named Mic34 and Mic40 (Figures 1 and 2B) that are peripheral to the IM and not membrane-anchored by a TMD-like conventional Mic60 in other eukaryotes [75], and even extant alphaproteobacteria [3]. Mic34 and Mic40 emerged early in evolution in the common ancestor of the euglenazoan taxon [75] that encompasses protists with disc-like cristae: trypanosomes and euglenids, free-living protists that are capable of photosynthesis [76]. Another seemingly unique property of trypanosomal MICOS is that its peripheral subcomplex also incorporates a thioredoxin-like subunit, which appears to play a currently undefined role in the oxidative folding of proteins that are imported into the IMS between the inner and outer mitochondrial membranes [31,77]. In yeast, proteins that are processed in this way include CHCH proteins, like Mic19 [78]. Despite these differences, trypanosomal MICOS engages in cross-talk with F_1_F_O_-ATP synthase, indicating the coordination of these two crista-shaping factors is fundamental for crista formation since LECA [79].

F_1_F_O_-ATP synthase dimers assume diverse configurations that conspicuously affect crista morphology throughout eukaryotes [4,34]. The U-shaped dimers of ciliates curl their tubular cristae [80]. The off-center dimers of euglenazoans such as *T. brucei* [38] are arranged into ribbons at crista rims [81], bestowing them with their distinctive discoidal appearance. The array of F_1_F_O_-ATP synthase dimers of the unicellular alga Polytomella also lends their cristae a disc-like shape [82], an instance of convergent evolution. Uniquely, these dimers localize to CJs as well as crista rims. Instead of dimers, *T. gondii* F_1_F_O_-ATP synthase forms cyclic hexamers at the tip of cristae, giving their cristae its distinctive bulbous shape [83]. In Toxoplasma, ATPase inhibitory factor 1, which normally prevents futile consumption of ATP by F_1_F_O_-ATP synthase during hypoxia [84], appears to be repurposed for controlling crista density [85]. The assorted dimer configurations discussed here, and ultimately the emergent crista shapes, are all variations of the F_O_ interface that was first discovered in yeast [36], and have diversified over time since LECA [38].

Structural investigation of protists has revealed that F_1_F_O_-ATP synthase (*i.e*. complex V) is not the only OXPHOS complex that contributes to crista morphogenesis. Super-complexes incorporating the electron transport chain (ETC) complexes also seem to confer membrane curvature, a role that is not apparent from the architecture of the mammalian respirasome, made up of NADH dehydrogenase (Complex I), a ubiquinol:cytochrome *c* oxidoreductase (Complex III) dimer, and cytochrome *c* oxidase (Complex IV), a configuration named I-III_2_-IV [86]. In ciliates, the semi-circularly shaped I-II-III_2_-IV_2_ megacomplex, which surprisingly includes succinate dehydrogenase (Complex II), likely curves the CM into tubular morphology [87,88]. Similarly, the III_2_-IV supercomplex of *T. gondii* displays a kinked interface that contributes to membrane curvature [89] (Figure 2C). In the euglenid *Euglena gracilis*, the I-III_2_-IV respirasome architecture appears to induce a negative curvature that aligns with a topological transition zone between the crista rims and the flat side of its discoidal cristae [90] (Figure 2B). The surprising II-IV_2_-V supercomplex, the first to combine a F_1_F_O_-ATP synthase monomer with other OXPHOS complexes, has been recently reported to cap F_1_F_O_-ATP synthase dimer ribbons at the discoidal-crista rims of the trypanosomatids *T. brucei* and *Leishmania tarentolae* while inducing positive membrane curvature [91,92] (Figure 2B) The F_1_F_O_-ATP synthase monomer is incorporated into II-IV_2_-V via a heterodimer of two subunit g-like proteins, and their depletion results in cristae transforming from discoidal to tubular morphology. In light of these findings, the planar architecture of the mammalian respirasome may annul membrane curvature at flat regions of the lamellar cristae, which are enriched with ETC machinery [93]. What is certain, investigation of protists has introduced the concept that respiratory super-complexes can also be vital crista-shaping factors.

The manner and degree to which CL shapes non-opisthokont cristae is as nebulous as it is in animal and fungal model organisms. CL permeates OXPHOS complexes observed at atomic resolution in euglenids [90] and ciliates [87,88,94]. The phospholipid is even found at the interaction interface between each F_1_F_O_-ATP synthase protamer within the euglenazoan dimer [38,95] and *T. gondii* hexamer [83]. This could explain why the downregulation of CL synthesis destabilizes OXPHOS complexes and deforms cristae [96,97], whereas this treatment has a nominal effect on the mitochondrial morphology and physiology of budding yeast [21]. How rearrangement of CL fatty acyl side chains (e.g. saturation and length) impacts crista shape remains an open question outside of opisthokonts as well as within. Adding to the mystery is the patchy distribution of Tafazzin, being present only in just a handful of diverse taxa that include opisthokonts, land plants, amoebae, and stramenopiles [98]. Therefore, can CL be remodeled *in situ* within the IM, perhaps resulting in altered membrane curvature, in the other lineages without Tafazzin?

## How do morphogenesis factors come together to build a crista?

Eukaryotes undergo cellular differentiation to create a pool of specialized cells (e.g. in multicellular animals and plants) or rearrange themselves into forms that are adapted to survive ecological bottlenecks (e.g. unicellular parasites). These massive changes are linked to different mitochondrial morphologies and are almost always accompanied by a rewiring of energy metabolism [99,100]: the general paradigm is that cells transition from glycolytic to OXPHOS-centered metabolism as they develop from pluripotent to specialized cells. We hypothesize that crista maturation, the biogenesis of an OXPHOS-competent crista from a precursor form or even *de novo*, underlies this metabolic switch.

**Figure 3:**
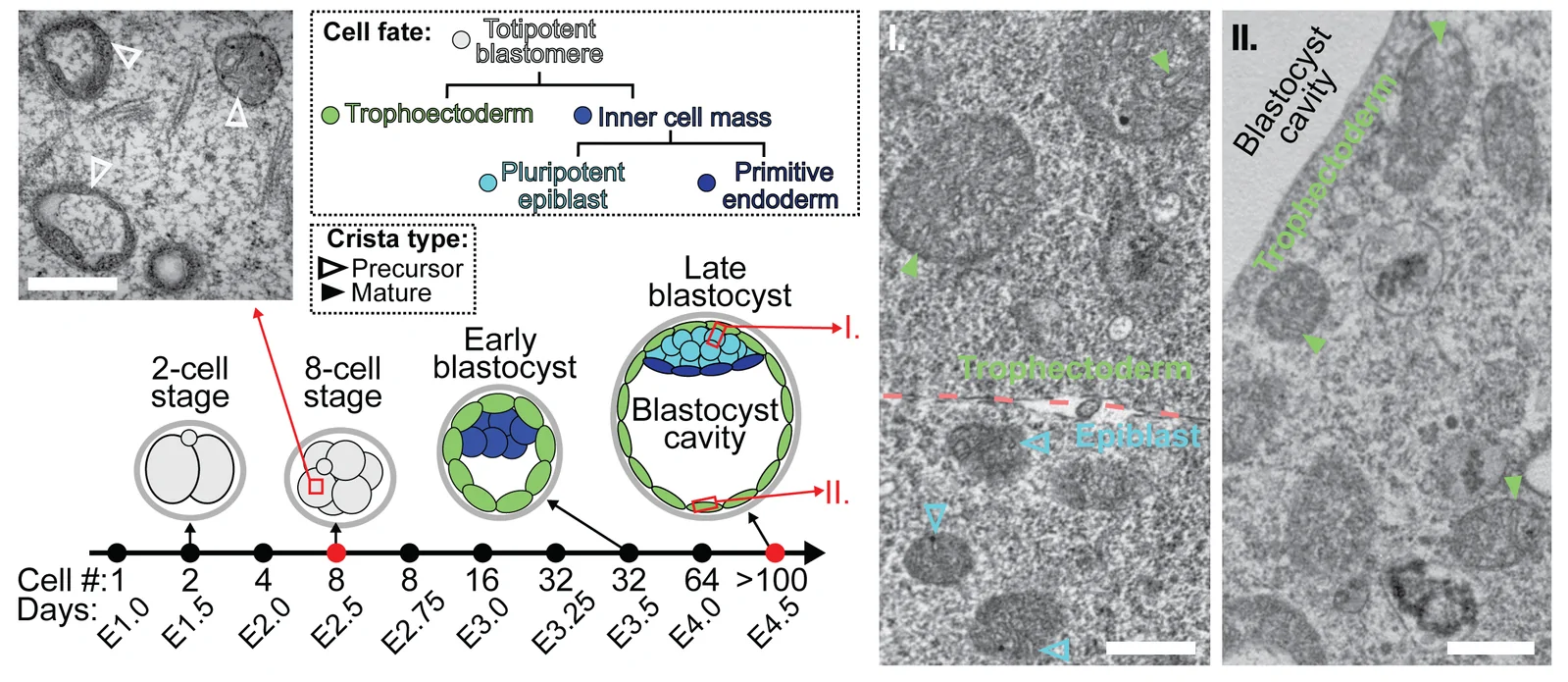
Atlas of cristae formation during preimplantation mouse embryo development A developmental timeline of preimplantation, adapted from Mihajlovic and Bruce (2017) [101], is shown with the emergence of the three color-coded blastocyst lineages (trophectoderm, primitive endoderm, epiblast; see ‘Cell fate’ panel). Matching solid and open arrowheads in the transmission electron micrographs indicate putative precursor mitochondria and mature, cristae-rich mitochondria (in the trophectoderm), respectively. Images were acquired as described [3,42,69] and correspond to stage-specific positions (E2.5 or E4.5) highlighted by red boxes in the schematic. At E4.5 (panel I), a red dashed line marks the plasma membrane separating polar trophectoderm and epiblast; scale bar: 500 nm.

Crista maturation appears to take on an underappreciated role in cell differentiation during early stages of mammalian development within the preimplantation embryo (Figure 3). During this process, three distinct cell lineages arise via two sequential steps [101,102], summarized in Figure 3. Drawing primarily from investigations in mice [103], early-stage embryos (*e.g.* E2.5) remain in a metabolically quiet phase with low OXPHOS, supporting developmental plasticity and limiting reactive oxygen species (ROS) [104,105]. This metabolic state manifests as spherical mitochondria sparsely decorated with immature cristae (Figure 3). However, as the trophectoderm and inner cell mass (ICM) lineages become specified, metabolism bifurcates [106,107]. The ICM retains rounded, cristae-poor mitochondria, relying predominantly on aerobic glycolysis to sustain pluripotency. Conversely, trophectoderm cells acquire elongated, cristae-rich mitochondria and exhibit high levels of OXPHOS [108]. This transition supports the trophectoderm’s higher energy requirements for differentiation and generating the osmotic gradient necessary to expand the blastocyst cavity. Consistently, disrupted expression of mitochondrially encoded genes, as normally induced by translocation of the key trophectoderm-specifying transcription factor TEAD4 into mitochondria, results in defects in cristae morphology in trophoblastic stem cell models [108]. This potentially links crista formation to OXPHOS assembly, an association also observed in yeast [12,29,30]. At the late blastocyst stage (E4.5), mitochondria in both ICM-derived lineages remain superficially underdeveloped relative to the trophectoderm (Figure 3).

After implantation into the uterine lining, the trophectoderm gives rise to cell lineages that comprise the embryonic component of the placenta, including the pluripotent cytotrophoblast that differentiates into syncytiotrophoblast cells [101,109]. As in the preimplantation cell lineages, their mitochondria exhibit morpholologically distinct cristae [109]. However, in contrast, the pluripotent cells display mature lamellar cristae whereas the cristae of the differentiated placental lineages appear to have regressed morphologically and biochemically, exhibiting lower levels of MICOS and respiratory chain supercomplexes.

The case of early mammalian embryogenesis highlights the need to understand the developmental program underlying crista formation. So far, this process remains a black box, occluding a holistic understanding of cell differentiation mechanisms. Is one of the aforementioned crista-shaping factors the main regulator of crista maturation? Is there a sequence of events that must be followed, e.g. does MICOS assembly precede the assembly of F_1_F_O_-ATP synthase dimer rows? Do different crista morphologies follow completely different routes or do they differ in the duration and/or overlap of each step? Are cristae formed by extrusion of the IM or by fusing a crista vesicle produced in the matrix to the IM [110]? These questions remain unanswered in opisthokonts and non-opisthokonts alike.

For now, there are a handful of studies that can inform future research on crista formation. In *S. cerevisiae*, two cristae formation pathways have been proposed that lead to either lamellar or tubular cristae, both of which require MICOS and F_1_F_O_-ATP synthase dimers [54]. Interestingly, lamellar cristae biogenesis involves Mgm1-mediated IM fusion, in contrast to Opa1’s autonomous crista remodelling activity [47,49].

Crista maturation has also been investigated during fruit fly [111] and cardiomyocyte development [68]. Mitochondria become activated in flight muscle cells after the adult fly emerges from the pupae, a process that involves lamellar crista formation and the assembly of the terminal ETC complex cytochrome *c* oxidase. Interestingly, some mature cristae were observed not to be connected to the IM, prompting the authors to propose that lamellar cristae could arise both from IM invagination as well as from vesicle germination in the mitochondrial matrix. During heart development, cardiomyocytes react to a fatty acid surge, which promotes crista detachment from the IM prior to increased crista formation, which eventually yields mitochondria that are densely packed with cristae [68]. Cardiomyocyte crista maturation is accompanied by supercomplex assembly and is dependent on the CL remodelling enzyme Tafazzin. From this small sample size, it appears that crista maturation in animals may involve crista detachment from the IM.

The unicellular parasites that cause malaria and sleeping sickness, plasmodium, and trypanosomes, have complex life-cycles that allow them to survive within their mammalian hosts and insect vectors. In both cases, mitochondria are activated in insect-dwelling stages [112,113]. Plasmodium cristae develop from apparently acristate mitochondria [72,114]. Crista maturation proceeds even in the absence of Mic60 or Mic19, although the cristae display the typical detached cristae phenotype. Surprisingly, the lack of CJs does not compromise *in vitro* growth or differentiation into insect-stage *P. falciparum*. Even though the pathogenic lifecycle stage of *T. brucei* that infects the mammalian bloodstream is incapable of OXPHOS [113], stub-like precursor cristae are still maintained in the mitochondrion [115]. This situation is reminiscent of the immature cristae found in earliest stages of mouse embryogenesis (Figure 3), suggesting crista maturation from precursor forms may be more common than their *de novo* biogenesis. The recent finding that MICOS is assembled on the precursor cristae of ‘bloodstream’ *T. brucei* suggests a pre-adaptation for crista maturation [116]. Ongoing research by our and other groups hopes to unravel the developmental program underlying trypanosomal crista maturation. Prior and future reports in various organisms may reveal common design principles shared by crista maturation pathways, which we postulate is a vital step in eukaryotic cell differentiation.

## General design principles underlying crista formation

**Figure 4:**
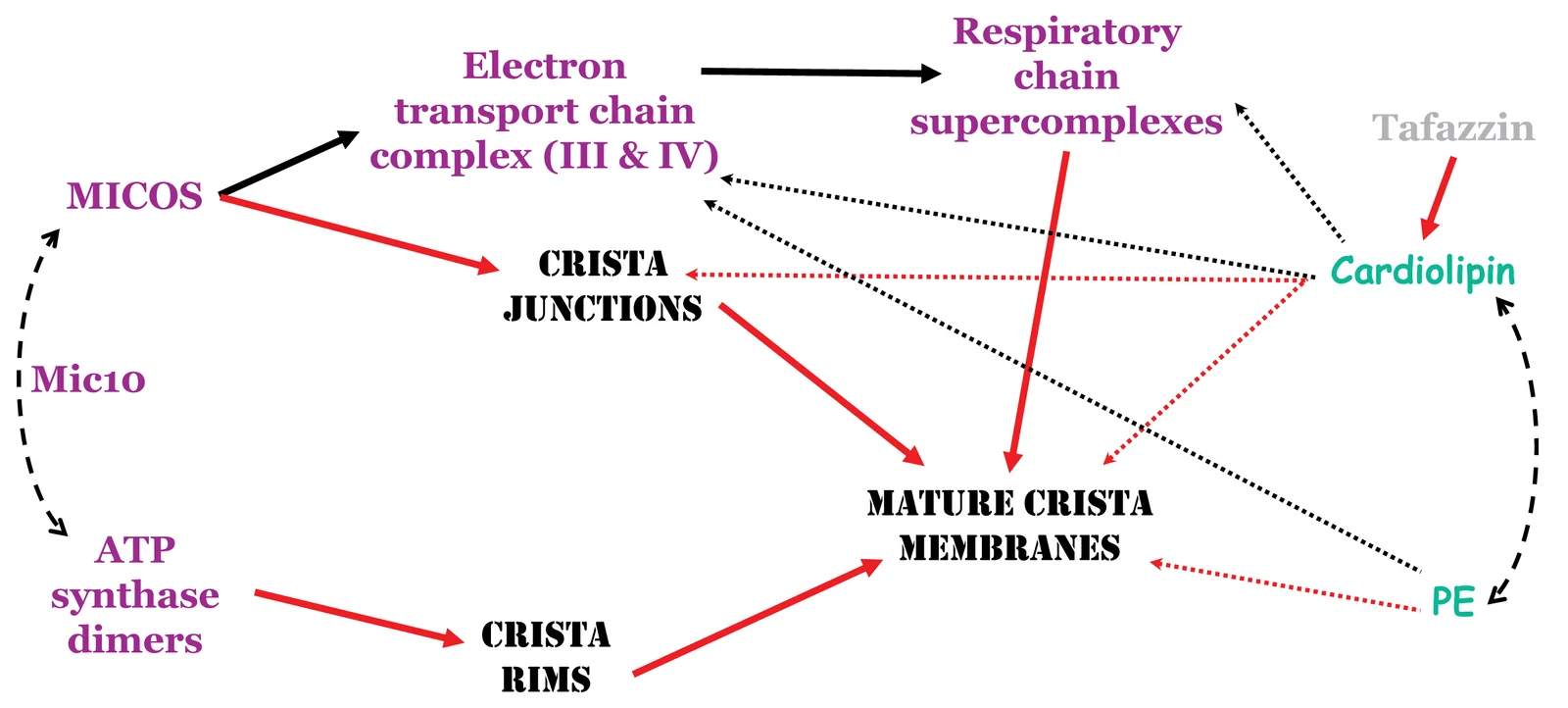
Hypothetical interdependencies of the factors involved in crista formation reviewed here Universally conserved factors besides Taffazin (grey) – whose distribution suggests it was likely in LECA – are shown in colors to depict specific elements: at the top and left are protein-composed (macro)molecules in purple; at the center are crista structures in black; on the right are non-bilayer phospholipids in cyan. Dashed double-headed arrows depict cross-talk. Solid and dotted arrows show protein and phospholipid activity, respectively. Red arrows indicate shaping/remodelling; black arrows indicate complex assembly. Opa1 and Mgm1 are not included here due to their restricted distribution (Figure 1).

By synthesizing information gained from organisms belonging to and excluded from the opisthokont lineage, some preliminary crista formation design principles emerge, which are summarized in Figure 4. The first is that MICOS and F_1_F_O_-ATP synthase dimers seem to be the main regulators of crista shape. Their cross-talk appears to be necessary for coordinating their activities on different parts of the crista. However, supercomplexes can also be recruited to induce or inhibit CM curvature. It is plausible that other molecules have evolved in specific clades to make novel contributions to crista morphogenesis. Opa1 and Mgm1 represent such lineage-specific factors. It remains to be seen if other dynamin-related proteins will act on the IM in a similar way [117]. Nevertheless, such evolutionarily ‘recent’ innovations would have to engage with more fundamental machineries to exert their action, e.g. Opa1 and MICOS. Finally, CL’s impact on crista formation remains a wildcard but appears to be non-trivial whether it shapes membranes directly and/or indirectly.

The study of non-opisthokont model organisms has clearly shown that divergent IM machineries and their distinctive organization contribute to a wide variety of cristae morphologies. The most recent structural works suggest that protists evolved to couple the bioenergetic activity of their OXPHOS complexes with a membrane-shaping function that is not found in mammalian or fungal mitochondria, which might reflect an adaptation strategy to compensate for the lack of other canonical membrane-shaping proteins such as Opa1, Mgm1, and/or canonical MICOS subunits. These organisms could help us develop an evolutionary framework for the study of crista biogenesis, as well as provide important clues to define the constraints of this process and the general mechanisms behind crista formation in all types of mitochondria.

## Perspectives

- The development of mitochondrial cristae from immature to fully functional forms underpins a switch to metabolism based on cellular respiration. This metabolic shift is important for cell differentiation in multicellular and unicellular eukaryotes alike.
- The molecules that shape mitochondria cristae are well known, with the most ubiquitous being the ATP synthase and MICOS multiprotein complexes plus respiratory chain supercomplexes, phospholipid CL, and dynamin-related proteins such as Opa1 and Mgm1.
- The current gap in knowledge is how these factors are coordinated during crista maturation. An ‘evo-devo’ approach involving investigation of diverse eukaryotes would identify general design principles that underlie crista formation.

## Open Access

Open access for this article is provided via our Subscribe to Open programme, made possible through the support of our subscribing institutions. Learn more here: https://portlandpress.com/pages/open_access_options_and_prices#conditional.

## Abbreviations

CJ: crista junction
CL: cardiolipin
CM: crista membrane
ETC: electron transport chain
IBM: inner boundary membrane
IM: inner membrane
IMS: intermembrane space
LECA: last eukaryotic common ancestor
OXPHOS: oxidative phosphorylation
TMD: transmembrane domain
