# Correction: The molecular mechanisms of crista formation: how mitochondria give themselves breathing room

**DOI:** 10.1042/BST20260167_COR

**Published:** 2026-09-08

**Authors:** Lilia Colina-Tenorio, Martina Bohuslavová, Alexander W. Bruce, Hassan Hashimi

**Affiliations:** 1 Department of Molecular Biology and Genetics, Faculty of Science, University of South Bohemia, České Budějovice, Czechia; 2 Institute of Parasitology, Biology Centre, Czech Academy of Sciences, České Budějovice, Czechia

**Keywords:** ATP synthase, cristae, dynamin-related protein, MICOS, mitochondria, oxidative phosphorylation

The authors of the original article titled “The molecular mechanisms of crista formation: how mitochondria give themselves breathing room” 10.1042/BST20260167 would like to correct an error within Figure 1, as described below.

**Figure 1:**
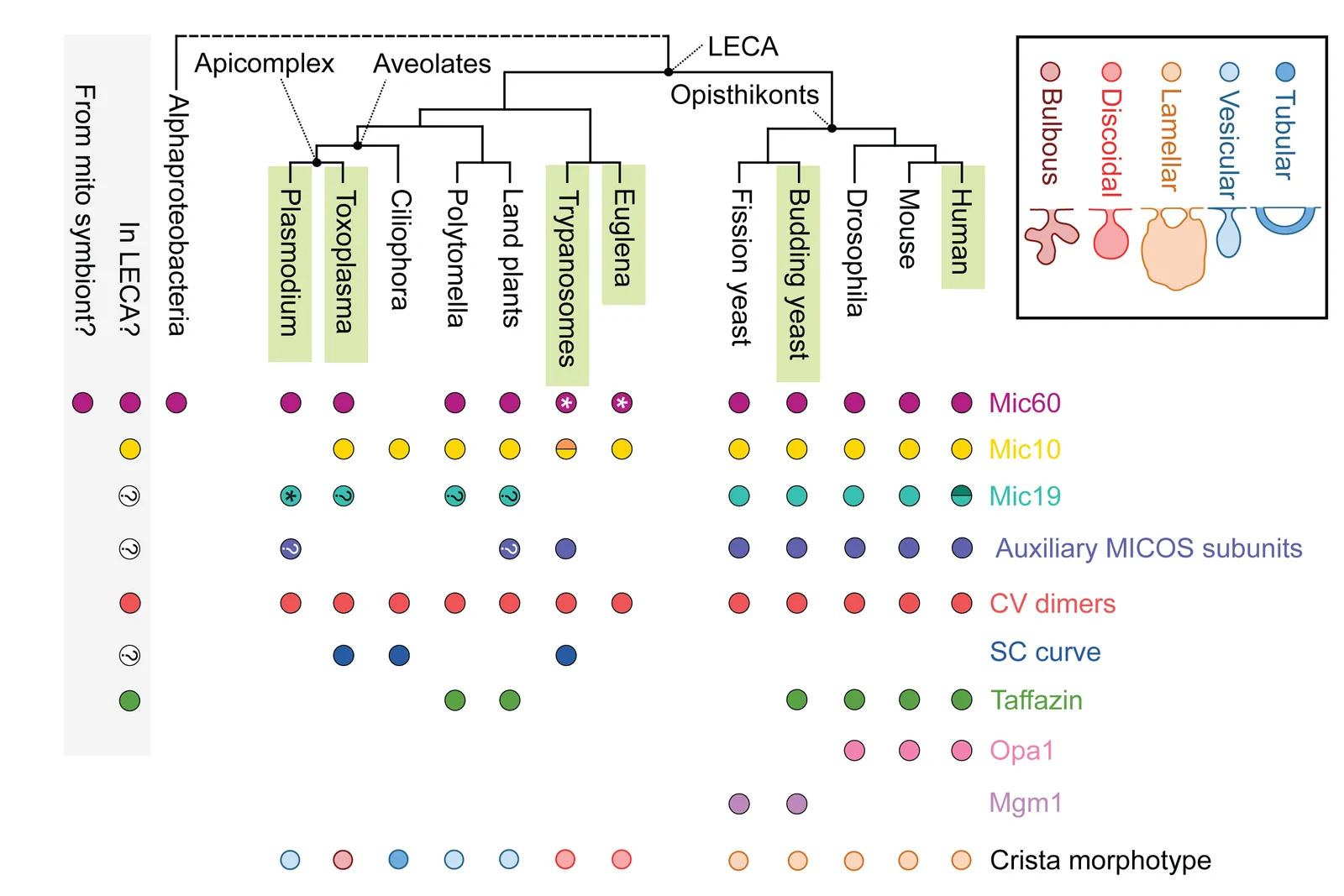
The phylogenetic distribution of crista morphotypes and selected crista formation factors in the organisms discussed in this review

Figure 1 mistakenly omitted the presence of FoF1 ATP synthase dimers (labelled as ‘CV dimers at the top), i.e. Toxoplasma gondii indeed possesses FoF1 ATP synthase dimers like other aerobic eukaryotes. Thus, this row should also bear a red circle. The authors apologize for this oversight and emphasize that this correction does not change the overall opinions and factual content of the review.

The requested correction has been assessed and agreed by the Editorial Board. The authors declare that this correction does not change the results or conclusions of their paper.

